# Acupuncture, chiropractic and osteopathy use in Australia: a national population survey

**DOI:** 10.1186/1471-2458-8-105

**Published:** 2008-04-01

**Authors:** Charlie CL Xue, Anthony L Zhang, Vivian Lin, Ray Myers, Barbara Polus, David F Story

**Affiliations:** 1 Division of Chinese Medicine, School of Health Sciences, World Health Organization Collaborating Centre for Traditional Medicine, RMIT University, Melbourne, Australia; 2 School of Public Health, La Trobe University, Melbourne, Australia; 3 Division of Osteopathy, School of Health Sciences, RMIT University, Melbourne, Australia; 4 Division of Chiropractic, School of Health Sciences, RMIT University, Melbourne, Australia

## Abstract

**Background:**

There have been no published national studies on the use in Australia of the manipulative therapies, acupuncture, chiropractic or osteopathy, or on matters including the purposes for which these therapies are used, treatment outcomes and the socio-demographic characteristics of users.

**Methods:**

This study on the three manipulative therapies was a component of a broader investigation on the use of complementary and alternative therapies. For this we conducted a cross-sectional, population survey on a representative sample of 1,067 adults from the six states and two territories of Australia in 2005 by computer-assisted telephone interviews. The sample was recruited by random digit dialling.

**Results:**

Over a 12-month period, approximately one in four adult Australians used either acupuncture (9.2%), chiropractic (16.1%) or osteopathy (4.6%) at least once. It is estimated that, adult Australians made 32.3 million visits to acupuncturists, chiropractors and osteopaths, incurring personal expenditure estimated to be A$1.58 billion in total. The most common conditions treated were back pain and related problems and over 90% of the users of each therapy considered their treatment to be very or somewhat helpful. Adverse events are reported. Nearly one fifth of users were referred to manipulative therapy practitioners by medical practitioners.

**Conclusion:**

There is substantial use of manipulative therapies by adult Australians, especially for back-related problems. Treatments incur considerable personal expenditure. In general, patient experience is positive. Referral by medical practitioners is a major determinant of use of these manipulative therapies.

## Background

The term complementary and alternative medicine (CAM) covers a diverse range of therapies, including various forms of herbal medicine, nutritional supplements, homeopathic medicines and a range of manipulative therapies. The main manipulative therapies generally considered to be complementary medicine are acupuncture, chiropractic and osteopathy. In all Australian states, chiropractic and osteopathy are subject to statutory regulation and, in the State of Victoria, non-medically qualified acupuncture practitioners are required to be registered by the Chinese Medicine Registration Board of Victoria. Acupuncturists, chiropractors and osteopaths undertake degree-level training in their disciplines and their services are covered by most major Australian private health insurance funds. Also, the Australian Government's Health Insurance Commission (Medicare) provides rebates for acupuncture services provided by approved medical practitioners. Over the last two years, acupuncture has been introduced as a routine clinical care option for patients with acute pain and other clinical conditions attending the Emergency Departments of two major Melbourne hospitals, the Northern Hospital and the Epworth Hospital.

Despite the apparent popularity of acupuncture, chiropractic and osteopathy in Australia, there have been no published national studies of these therapies in regard to the prevalence of their use, patient expenditure, the demographic characteristics of users, the medical conditions for which they are used, the frequency of referral to practitioners by other health-care professionals, perceived benefits and adverse effects of treatment. To investigate these and other matters for a much broader range of CAM therapies in Australia we conducted a national population survey in 2005 [1]. The purpose of the present paper is to report the detailed findings from the survey in respect of acupuncture, chiropractic and osteopathy.

## Methods

Ethical approval for the study was obtained from the Royal Melbourne Institute of Technology (RMIT) University's Human Research Ethics Committee.

### Survey Design

An Australian population survey, conducted in 2002 in the state of South Australia, indicated that over 50% of the Australian population used some form of CAM over a 12 months period [2]. For our broad survey on CAM use we estimated (Epi Info 6.0 [3]) that a sample of 1,067 interviews would yield prevalence data on CAM use overall, with 95% confidence internals of plus/minus three percent. During May and June 2005, using random digit telephone dialling, we recruited this number of adult Australian (18 years or older), from all Australian states and territories, for a computer-assisted telephone interview (CATI) [1,4]. In an attempt to obtain a representative sample, national quotas for gender and defined age groups were allocated according to Australian Bureau of Statistics (ABS) 2005 survey data [5]. A maximum of 15 attempts were made to establish contact with an individual. On first contact, the member of the household with the next birthday was asked to participate in the survey. If the person was not immediately available, an appointment was made for a subsequent interview. If necessary, up to 10 additional attempts were made to interview the selected individual. No financial incentive was provided.

Participants were first asked whether or not they had used each of 17 common forms of CAM in the preceding 12 months, including acupuncture, chiropractic and osteopathy. If respondents had used a specific form of CAM, they were then asked if they had visited a practitioner of that form of CAM in the 12 month period. Those that had used one or more of the three manipulative therapies were asked a series of questions covering specific matters related to the manipulative therapies that they had used.

### Statistical analyses

Data were weighted to adjust for any deviation in sampling from ABS stratified population data in regard to state/territory of residence, gender and age [5]. 95% Confidence intervals (CI) were calculated for prevalence data for each socio-demographic category and chi-square tests were used to compare differences. Factors found to be statistically significant at the univariate level were entered into a multivariate logistic regression model to reveal the association (odds ratio) between the use of acupuncture, chiropractic and osteopathy and the variables. Probability levels less than 0.05 were taken to indicate statistical significance. All analyses were conducted using the Statistical Package for Social Sciences (SPSS) for Windows, version 15.0.

## Results

A total of 1,067 individuals completed the survey with an average interview length of 13.5 minutes. The participation rate was approximately 15% [1]. Statistical comparisons show that basic demographic data for the study population in respect of state of residence, gender, age-range, self-reported health status, Australian/overseas born status, education status, employment status and household income were comparable to national census and relevant national health survey data [1,5].

### Prevalence of use and practitioner visits

Approximately one in four (24.5%, 95% CI: 21.9% – 27.1%, being 271 of the 1,067 interviewees) reported using at least one of the three manipulative therapies in the previous 12 months. Chiropractic was used by 16.1% (n = 176) of the survey participants, acupuncture by 9.2% (n = 101) and osteopathy by 4.6% (n = 51). Due to the provider nature of the therapies, the majority of the users had visited practitioners to receive treatment. Thus, 21.2% (95% CI: 18.8% – 23.7%) of survey participants had visited a practitioner of at least one of the three forms of manipulative therapies. The proportions of survey participants that made practitioner visits for the individual therapies were 7.5% (95% CI: 5.9% – 9.1%) for acupuncture, 14.6% (95% CI: 12.4% – 16.7%) for chiropractic and 3.5% (95% CI: 2.4% – 4.6%) for osteopathy. A small proportion of the users of each therapy indicated that they did not receive their treatment from a practitioner of the therapy.

The average numbers of visits to practitioners by users of the three therapies over the 12-month period were 8.8 for acupuncture, 8.4 for chiropractic, and 5.7 for osteopathy. Hence, based on a national adult population of 15.5 million, it was estimated that Australian adults had made 10.2, 19.1 and 3.1 million visits to acupuncturists, chiropractors and osteopaths, respectively in the 12 month period. The total number of visits to the three types of therapists was 32.3 million (95% CI: 26.0 million – 38.6 million), representing almost half of all visits to all types of CAM practitioners in the survey.

### Socio-demographic representation of users

The representation of users of acupuncture, chiropractic and osteopathy in our sample in various socio-demographic categories are presented in Table 1. The only significant gender difference was for osteopathy, there being a higher proportion of female than male users (*p *< 0.05, Z-test). There were no significant differences in the prevalence of use of any of the three therapies between the three age ranges investigated (Table 1). A significantly higher proportion of participants with private health insurance had used chiropractic than participants without private health insurance (*p *< 0.05, Z-test; Table 1). Neither self-reported health status nor employment status (employed/unemployed) seemed to be a determinant of the prevalence of use of any of the three therapies (Table 1).

**Table 1:** Use of acupuncture, chiropractic and osteopathy by participants in various socio-demographic categories

	**Percentage (95% confidence interval)**		
	
**Characteristic**	**Acupuncture**	**Chiropractic**	**Osteopathy**
**Gender**			
Female	9.6 (7.1 – 12.1)	17.1 (13.9 – 20.3)	5.8 (3.8 – 7.8)
Male	8.9 (6.4 – 11.3)	15.0 (12.0 – 18.1)	3.3 (1.8 – 4.8)
**Age (year)**			
18–34	7.6 (4.8 – 10.5)	15.0 (11.2 – 18.9)	3.1 (1.3 – 5.0)
35–64	10.3 (7.7 – 12.8)	16.7 (13.6 – 19.9)	5.0 (3.2 – 6.8)
65+	9.2 (5.1 – 13.4)	15.7 (10.5 – 21.0)	5.9 (2.5 – 9.3)
**Country of birth**			
Australia	10.2 (8.1 – 12.3)	18.1 (15.5 – 20.8)	4.8 (3.4 – 6.3)
Overseas	6.5 (3.4 – 9.5)	9.8 (6.1 – 13.5)	3.8 (1.4 – 6.1)
**Self-reported health status**			
Excellent/very good/good	8.6 (6.8 – 10.5)	15.2 (12.8 – 17.5)	4.5 (3.2 – 5.9)
Fair/poor	12.1 (7.1 – 17.2)	20.6 (14.4 – 26.9)	4.8 (1.5 – 8.1)
**Post-secondary education**			
No	6.4 (4.2 – 8.7)	15.4 (12.1 – 18.8)	3.6 (1.9 – 5.3)
Yes	11.3 (8.8 – 13.8)	16.7 (13.8 – 19.7)	5.3 (3.6 – 7.1)
**Employment**			
Employed	9.8 (7.6 – 12.0)	16.8 (14.0 – 19.6)	4.5 (2.9 – 6.0)
Unemployed/not in work force	7.8 (5.0 – 10.5)	14.5 (10.9 – 18.1)	4.7 (2.5 – 6.9)
**Private health insurance**			
Yes	10.8 (8.3 – 13.3)	19.0 (15.8 – 22.1)	5.6 (3.7 – 7.4)
No	7.4 (5.0 – 9.8)	12.4 (9.4 – 15.4)	3.3 (1.7 – 5.0)
**Annual household income**			
<A$20,000	8.1 (3.8 – 12.3)	7.3 (3.3 – 11.4)	6.7 (2.8 – 10.6)
A$20,000 – A$40,000	7.0 (3.5 – 10.5)	19.0 (13.6 – 24.4)	1.3 (-0.3 – 2.9)
>A$40,000	10.1 (7.6 – 12.6)	18.4 (15.2 – 21.6)	5.5 (3.7 – 7.4)
**Australian state of residence**			
New South Wales	9.9 (6.8 – 12.9)	17.0 (13.1 – 20.9)	6.0 (3.5 – 8.4)
Victoria	10.9 (7.2 – 14.6)	17.4 (12.8 – 21.9)	5.2 (2.6 – 7.9)
Queensland	11.6 (7.3 – 16)	14.9 (10.0 – 19.7)	3.8 (1.2 – 6.5)
South Australia	6.9 (1.4 – 12.3)	17.0 (8.9 – 25.1)	1.2 (-1.1 – 3.5)
Western Australia	2.7 (-0.4 – 5.8)	14.4 (7.7 – 21.1)	2.2 (-0.6 – 5.0)

### Differences between Australian states

As can be seen from Table 1, there are marked differences in the prevalence of use of acupuncture and osteopathy between residents of different Australian states. Only 2.7% of participants from Western Australia were acupuncture users, there being approximately four times higher rates in New South Wales, Victoria and Queensland. For osteopathy, the prevalence of use in New South Wales was about four-fold that in both South Australia and Western Australia. In contrast to acupuncture and osteopathy, the use of chiropractic appears to be consistent across the mainland states of Australia (Table 1).

### Socio-demographic correlations

Logistic regression analyses of the survey data revealed some significant correlations between a number of socio-demographic factors and the prevalence of use of the three manipulative therapies (Table 2). Those participants with post-secondary education were more likely to have used acupuncture than those without post secondary education and those in the two highest household income brackets were more likely to have used chiropractic than those in the lowest income bracket. In contrast, osteopathy was used more commonly by those in the lowest income bracket. It is also noteworthy that a higher proportion of Australian-born participants had used chiropractic than those born overseas.

**Table 2:** Logistic regression analyses of factors associated with the use of acupuncture, chiropractic and osteopathy

**Factors**	Acupuncture Odds ratio (95% CI*)	Chiropractic Odds ratio (95% CI*)	Osteopathy Odds ratio (95%CI*)
Post-secondary education			
No	1.0	...	...
Yes	1.7 (1.0–2.7)	...	...
Income range			
<A$20,000	...	1.0	1.0
A$20,000–A$40,000	...	3.2 (1.5–7.3)	0.1 (0.0–0.4)
>A$40,000	...	3.8 (1.8–7.8)	0.6 (0.3–1.3)
Country of birth			
Overseas	...	1.0	...
Australia	...	2.1 (1.2–3.4)	...
Consulted medical doctor in past 12 months			
No	1.0	1.0	...
Yes	2.1 (1.0–4.2)	2.0 (1.1–3.5)	...
Visited medical doctor for back problems			
No	...	1.0	1.0
Yes	...	3.5 (1.4–8.4)	4.3 (1.1–16.4)
Omnibus Tests of Model Coefficients χ^2^-test (degrees of freedom, *p *value)	23.04 (3, < 0.0001)	60.24 (6, < 0.0001)	34.82 (4, < 0.0001)

*95% confidence interval

As also shown in Table 2, more participants who had consulted a medical practitioner in the preceding 12 months used acupuncture and chiropractic than those who had not consulted a medical practitioner. There was no such association between medical practitioner consultations and use of osteopathy. However, perhaps not surprisingly, survey participants who had consulted a medical practitioner specifically for back-related problems were more likely to have also used chiropractic and osteopathy than those who had not sought medical treatment for back-related problems (Table 2).

### Rationale of use

Reasons that participants in our survey chose to use each of the three manipulative therapies are summarised in Table 3. The majority of those who used acupuncture (92.0%) did so to treat a specific medical condition, the most frequent being back pain and related problems, and shoulder pain and related problems.

**Table 3:** Reported main reasons for using acupuncture, chiropractic and osteopathy

Rationale of use*	**Percentage using therapy for specified purpose**		
	
	**Acupuncture (n = 101)**	**Chiropractic (n = 176)**	**Osteopathy (n = 51)**
**1. Treatment of a specific medical condition**	92.0	68.6	75.9
Five most common complaints:			
Back pain/related problem	20.7	65.7	48.4
Shoulder pain/related problem	15.5	5.3	22.9
Arthritis	8.5	...	...
Injury	7.0	...	5.2
Knee problem	5.1	...	...
Neck pain/related problem	...	20.7	10.7
Headache and migraine	...	9.3	...
Non-specific musculoskeletal problem	...	14.9	35.2
**2. General health and well-being**	22.1	32.3	40.6
**3. Improving ability to undertake daily activity**	Not asked	36.2	41.9
**4. Improve sporting performance**	Not asked	9.4	17.1

*Users permitted to report multiple reasons of use

More than two thirds of chiropractic and osteopathy users indicated that their use of the therapy was to treat a specific medical condition. For chiropractic users, back pain and related problems, neck pain and related problems, and non-specific musculoskeletal problems were the most frequent conditions for which treatment was sought. For osteopathy, back pain and related problems, non-specific musculoskeletal problems and shoulder pain and related problems were the most frequent conditions nominated.

Enhancement of general health and well-being was nominated as a reason for treatment by about 22% of acupuncture users and by somewhat higher proportions of chiropractic and osteopathy users (approximately 32% and 41%, respectively). Well over a third of those who sought treatment from a chiropractor or osteopath did so to improve their ability to undertake normal daily activities. Somewhat lower proportions of chiropractic and osteopathic users chose the therapy to improve their sporting performance (Table 3).

### Outcomes of acupuncture, chiropractic and osteopathy

Acupuncture, chiropractic and osteopathy users were asked about their perceptions of the outcome of their treatment. Table 4 summarises the findings. Large majorities of users of each therapy were positive about the outcome of their treatment, reports of relief of pain and other symptoms being particularly prominent. Acupuncture users were asked if their treatment had resolved their disease/problem, and over 42% responded affirmatively. Substantial proportions of users of all three therapies considered that their treatment had improved their well-being (about 78% for acupuncture and 46% for both chiropractic and osteopathy). Overall, more than 90% of the users of each therapy believed that the manipulative treatments they chose were very helpful or somewhat helpful (Table 4).

**Table 4:** Perceived outcomes of acupuncture, chiropractic and osteopathy use

	**Percentage of users**		
	
**Outcome**	**Acupuncture (n = 101)**	**Chiropractic (n = 176)**	**Osteopathy (n = 51)**
Overall helpfulness*			
Very helpful	60.0	71.4	64.2
Somewhat helpful	30.1	21.6	28.4
Less helpful	7.8	5.0	3.0
**Positive outcomes**^†^			
Relieved pain/symptoms	87.1	71.9	79.5
Improved well-being	78.2	45.5	45.6
Cured the disease/solved the problem	41.6	Not asked	Not asked
Improved ability to undertake daily activities	Not asked	50.2	60.5
Decreased disability	Not asked	23.1	40.6
**Adverse events**^#^	3.0	4.0	7.8

*Some users did not respond to this question.

^†^Users permitted to report multiple positive outcomes.

# % of users who reported an adverse event

Adverse events with the manipulative therapies were minor and relatively rare. Among the 101 acupuncture users, three experienced pain after needling and one complained of bruising at needling sites. Seven of the 176 chiropractic users reported that they had experienced adverse events of treatment. Three reported post-treatment pain, two reported headache/migraine, one reported tiredness and one user considered that he/she had sustained a back injury from their treatment but this had not been confirmed. Four of the 51 osteopathy users reported adverse events. These included increased pain, soreness, tiredness and "neck cracks" after treatment.

### Referrals to manipulative therapists

Most commonly, acupuncture, chiropractic and osteopathy treatments were sought on the basis of recommendations by a friend or relative (40.8% of acupuncture users, 43.6% of chiropractic users and 38.1% of osteopathy users). The second most common referral was by medical practitioners (acupuncture 20.7%; chiropractic 20.0%, osteopathy 16.1%). Nearly one fifth of acupuncture (18.7%) and osteopathy (21.5%) users were referred to practitioners of these therapies by other CAM practitioners. The proportion of chiropractic users referred by other CAM practitioners was much lower (7.2%).

All survey respondents, regardless of whether or not they had previously used one or more of the three manipulative therapies, were asked if they would consider using any of them in the future. Nearly two thirds responded that they would consider the use of acupuncture (62.4%) and chiropractic (68.1%) while about half (52.0%) indicated that they would consider using osteopathy. Relatively high proportions of those who had used each manipulative therapy in the preceding 12 months indicated that they would consider using it again, the proportions being 94.7%, 93.3% and 87.6% for acupuncture, chiropractic and osteopathy, respectively.

## Discussion

In Australia, acupuncture, chiropractic and osteopathy are generally regarded as complementary therapies. Each involves services provided by practitioners in contrast to many other forms of CAM therapy which are self-selected and self-administered. The majority of users choose acupuncture, chiropractic and osteopathy to manage pain and specific medical conditions, back pain and related problems being particularly prominent considerations. All chiropractic and osteopathy practitioners receive degree-level training and the majority of acupuncture practitioners receive either university training or complete courses accredited by a national professional association. Each discipline is subject to regulation by either a statutory body and/or a national professional association (see below). Acupuncture treatments may be provided by accredited medical practitioners and by accredited non-medical personnel. From a patient's perspective the main difference between receiving acupuncture from a medical practitioner and a non-medical acupuncturist is the availability of government universal health insurance (Medicare) rebates when the acupuncture is provided by an accredited medical practitioner. The number of acupuncture services rebated by Medicare in 2005/2006 was 607,349 [6], which is only about 6% of the total number of annual acupuncture services estimated in our survey.

Previous studies on the use of much broader ranges of CAM therapies found that certain socio-demographic factors were correlated with CAM use, such as gender (CAM being more popular with females than males), age (the highest rates of CAM use being by the middle-aged), and household income and level of education (both being positively correlated with CAM use) [7,8]. Some of these associations also appear to be applicable to the use of one or more of the three complementary manipulative therapies investigated in the present study. Thus, osteopathy was considerably more popular amongst females than males but there was no gender difference in the use of acupuncture or chiropractic services. The prevalence of chiropractic use was significantly higher among those with household incomes more than A$20,000 per annum. In contrast, osteopathy appears to be more popular amongst those with household incomes less than A$20,000. In regard to level of education, the only association observed was that acupuncture was more commonly used by those with higher (post-secondary) education. A recent large-scale longitudinal study on women's health, found that middle-aged Australian women living in non-urban areas and women with a lower level of education were more likely to use chiropractic or osteopathy [9]. However, our data do not allow us to draw such conclusions.

We found that chiropractic was considerably more popular amongst those born in Australia than those born overseas. However, this was not so for acupuncture or osteopathy. It seems likely that the greater prevalence of use of chiropractic by those born in Australia than by those born overseas is due to the profession being well established in Australia, with university trained practitioners and being subject to statutory regulation, whereas in many other countries, particularly Asian countries, chiropractic is a less well known health-care service. It is worth noting that osteopathic medicine is not generally considered as a form of CAM in the US and thus has been excluded from most CAM population studies conducted in the US [8].

It is of interest that users of acupuncture and chiropractic in our survey tended to have also consulted a medical practitioner during the 12 month period investigated. In the case of osteopathy, there was not a significant correlation between osteopathy use and medical practitioner visits *per se*; however, both osteopathy and chiropractic users with back problems were highly likely to have also visited a medical practitioner. Similar to findings reported in the US [10] and from a previous Australian study [11], we found that acupuncture, chiropractic and osteopathy were most commonly used to treat chronic somatic and musculoskeletal conditions. Although our data do not allow us to draw conclusions about the clinical benefits of the manipulative therapies, a large majority of users of acupuncture, chiropractic and osteopathy considered that their treatment was effective, particularly in respect of relief of pain and other symptoms of their condition. It is also of interest that substantial proportions of the respondents who used each of the three manipulative therapies did so with the objective of enhancing their general health and well-being.

There have been no previous studies that have investigated the popularity of acupuncture, chiropractic and osteopathy in individual Australian states. Our data indicate that the prevalence of use of chiropractic is consistent in all five mainland states and our estimate of the prevalence of chiropractic use in South Australia (17.0%) is in agreement with that of a 2004 study (16.7%) [12]. In contrast to chiropractic, the popularity of acupuncture and osteopathy across Australia is much more variable. It is particularly striking that acupuncture and osteopathy were used by relatively low proportions of the Western Australians and South Australians in our sample, a situation that is likely to be due to there being relatively low numbers of practitioners of the two therapies in these states [13].

The substantial use of acupuncture, chiropractic and osteopathy by adult Australians involves considerable personal expenditure by users. Although rebates for acupuncture, chiropractic and osteopathy services are available from most private health insurance funds, the maximum annual rebates are usually limited. Acupuncture services by accredited medical acupuncturists are rebated by Medicare, although the number of services for which such rebates were provided is relatively low (see above). Practitioner fee structures are complex, there usually being a lower fee for a return visit to practitioners. Based on advice from the relevant professional associations, the fee for an acupuncture consultation is in the range $AU 35 – 50, that for chiropractic, $AU 35 – 60 and that for osteopathy, $AU 60 – 100. Taking the means of these ranges, from our survey findings, we estimate that the total annual personal expenditure nationally, before insurance rebates, for acupuncture, chiropractic and osteopathy services are $AU 432 million, $AU 905 million and $AU 246 million, respectively. Obviously, the limits set by health insurance funds for rebate might have impact on the frequency of use of these therapies. Given the limitation of current sample size, a larger national survey would be necessary to confirm these projections of expenditure.

The adverse events reported by users of acupuncture and chiropractic in our study were mild and low in frequency, with only one event, in chiropractic, considered (by the patient) as severe. The most frequent complaint by acupuncture users was mild needling pain; however, given that skin penetration is inherent in this therapy, such a subjective sensation should probably not be considered to be an adverse event. There was a somewhat higher frequency of adverse events reported by osteopathy users; however, some caution needs to be exercised in extrapolating this observation, due to the relatively low number of osteopathy users in the study. The safety profiles of these three manual therapies based on the user-reported events may also be partially explained by the fact that these professions are under statutory regulation in Australia (Victoria only for acupuncture). One of the main objectives of practitioner registration in Australia is the maintenance of standards of clinical practice, consistent with public safety.

Health-care providers now operate in a multi-disciplinary environment, which includes many forms of CAM practice. However, poor communication between practitioners of both conventional medicine and CAM therapies and their patients about the totality of their treatment and treatment options is a common finding of surveys of CAM use conducted in Australia [12] and other countries [14]. It has been reported that, frequently, patients do not disclose their use of CAM to their medical practitioner on the assumption that the "doctor does not need to know" and that "the doctor did not ask" [15]. This communication gap is certainly not in the best interests of patients or the providers. On the other hand, it is encouraging that our findings indicate that one in five users of acupuncture and chiropractic, were referred to a practitioner of these therapies by medical practitioners. Indeed it has been reported that chiropractic and acupuncture were the most frequently referred CAM modalities in the US [16]. Further, it has been estimated that approximately one in six medical general practitioners in Australia use acupuncture in their day-to-day practice (15.1%) [17].

There are several limitations to be considered in interpreting the findings of our study. Firstly, we achieved a participation rate of approximately only 15%, and therefore, there is a possible non-response bias. However, we consider that, by employing a rigorous sampling strategy, we obtained a sample representative of the target population [1]. Secondly, there were relatively small numbers of users of acupuncture, chiropractic and osteopathy in our survey sample of 1,067.

## Conclusion

We consider that our study provides reasonably accurate data on the prevalence of use of the three most common manipulative therapies and on the determinants and consequences of their use. Our findings indicate that approximately one in four adult Australians used acupuncture, chiropractic or osteopathy in 2005.

Frequently the manipulative therapies are used in conjunction with conventional medical treatments, a finding which highlights the need for better communication between patients, medical practitioners and CAM service providers. Also, it is suggested that another priority is further research, designed to explore in detail the consequences of the combined use of the complementary therapies and conventional medical treatments, particularly where they are being used for the same disease/condition.

## Competing interests

The author(s) declare that they have no competing interests.

## Authors' contributions

The following authors contributed to the conceptualisation of the project: CX, ALZ, VL, RM, BP and DFS. The following authors contributed to the data collection, analysis and interpretation: CX, ALZ and DFS. The following authors contributed to the draft of the manuscript: CX, ALZ and DFS. The following authors contributed to the review and commentary on the manuscript: VL, RM and BP. All authors read and approved the final manuscript.

## Pre-publication history

The pre-publication history for this paper can be accessed here:


